# The awareness of radiologists for the presence of lateral lymph nodes in patients with locally advanced rectal cancer: a single-centre, retrospective cohort study

**DOI:** 10.1007/s00330-022-08840-1

**Published:** 2022-05-18

**Authors:** T. C. Sluckin, Y. F. L. Rooker, S. Q. Kol, S. J. A. Hazen, J. B. Tuynman, J. Stoker, P. J. Tanis, K. Horsthuis, M. Kusters

**Affiliations:** 1 Department of Surgery, Amsterdam UMC location Vrije Universiteit Amsterdam, de Boelelaan 1117, Amsterdam, the Netherlands; 2 Cancer Center Amsterdam, Treatment and Quality of Life, Amsterdam, the Netherlands; 3 Cancer Center Amsterdam, Imaging and Biomarkers, Amsterdam, the Netherlands; 4 Department of Radiology and Nuclear Medicine, Amsterdam UMC location Vrije Universiteit Amsterdam, de Boelelaan 1117, Amsterdam, the Netherlands; 5 Department of Radiology and Nuclear Medicine, Amsterdam UMC location University of Amsterdam, Meibergdreef 9, Amsterdam, the Netherlands; 6 Department of Surgical Oncology and Gastrointestinal Surgery, Erasmus MC, Rotterdam, the Netherlands; 7 Department of Surgery, Amsterdam UMC location University of Amsterdam, Meibergdreef 9, Amsterdam, the Netherlands

**Keywords:** Lateral lymph nodes, Rectal cancer, MR-imaging

## Abstract

**Objectives:**

Enlarged lateral lymph nodes (LLNs) are associated with increased (lateral) local recurrence rates. Size and anatomical location should therefore always be reported by radiologists and discussed during multidisciplinary meetings. The objective was to investigate how often LLNs are mentioned in MRI reports in a tertiary referral centre.

**Methods:**

A single - centre, retrospective study of 202 patients treated for primary rectal cancer between 2012 and 2020, with at least a T2 tumour located within 12cm of the anorectal junction. The radiology reports were written by 30–40 consultant radiologists. MRI scans were independently re-assessed by an expert radiologist. The primary outcome was how often the presence or absence of LLNs was mentioned in the initial report.

**Results:**

Primary MRI reports explicitly mentioned the presence or absence of LLNs in 89 (44%) cases. Of the 43 reports with present LLNs, only one (1%) reported on all features such as size, location or malignant features. Expert review revealed 17 LLNs which were ≥ 7 mm (short-axis); two of these were not mentioned in the original reports. In 14/43 (33%) cases, LLNs were discussed during the primary multidisciplinary meeting, while 17/43 (40%) restaging MRI reports failed to report on the previously visible LLN. Reporting LLNs increased significantly with higher N-stage (*p* = .010) and over time (*p* = .042).

**Conclusions:**

Though improving with time, there is still limited consistency in reporting LLNs. Only 44% of primary MRI reports mentioned LLNs and relevant features of those LLNs were seldomly reported. Given the importance of this information for subsequent treatment; increased awareness, proper training and the use of templates are needed.

**Key Points:**

*• Comprehensive reporting of lateral lymph nodes in primary MRI reports was limited to less than 50%.*

*• Lateral lymph nodes are not always discussed during primary multidisciplinary meetings or mentioned in restaging reports.*

*• Improvements in the awareness and knowledge of lateral lymph nodes are needed to ensure adequate multidisciplinary treatment decisions.*

## Introduction

Magnetic resonance imaging (MRI) is uniformly considered the primary imaging modality for diagnosis and staging of (locally advanced) rectal cancer (LARC) [1–5]. For patients with mid- to low-LARC staged as at least cT3+ or N+, the universally accepted standard treatment is neoadjuvant (chemo)radiotherapy ((C)RT) followed by a total mesorectal excision (TME) [6]. This combination has reduced the 5-year local recurrence (LR) rate to 5–10% [7–9].

Low LARCs have an increased chance of spreading to lymph nodes located in the lateral pelvic compartments, lateral lymph nodes (LLNs), which are not resected during TME surgery [8, 10]. Nodal imaging research has until now, primarily focused on mesorectal lymph nodes, where data suggests that size alone has limited sensitivity and specificity. Hence, additional malignant features, such as internal heterogeneity, round shape and an irregular border, were added to size to increase diagnostic accuracy [11, 12]. However, recent evidence suggests that LLNs behave differently compared to mesorectal lymph nodes. An international retrospective study, including 1216 patients with MRI re-review, has shown that approximately 16% of patients with low LARC have an enlarged LLN (≥ 7 mm short-axis) on the primary MRI [13]. In that study, size and anatomical location of LLNs on primary and restaging imaging were significantly related to an increased LR risk. LLNs with a primary size of ≥ 7 mm had a 5-year lateral LR (LLR) rate of 19.5%. Those located in the internal iliac compartment which remained > 4 mm after (C)RT had a 5-year LLR rate of 52.3%. Obturator LLNs > 6 mm on the restaging MRI had a 17.8% 5-year LLR risk. Remarkably, morphological criteria were not found to be associated with these increased LLR rates [13]. These findings are supported by other studies in which LLNs ≥ 10 mm result in a 5-year LR rate of 30–40% [14–17]. These results emphasise that size and anatomical location of LLNs, and not morphological criteria, significantly influence the (L)LR risk.

A lateral lymph node dissection (LLND) can be performed in an attempt to reduce the lateral LR risk [14, 18, 19]; a procedure in which all lymphatic tissue is removed from the lateral compartments. Ogura et al [13, 20] found that performing an LLND in cases of persistently enlarged LLNs decreased the 5-year LR rate from 20–53% to 5–8%. This procedure is, however, not without risks of urinary and/or sexual dysfunction [14, 21–24]. Performing an LLND should therefore be based on the selection of ‘high-risk’ patients for whom the benefits of an LLND outweigh the risks.

Considering the significance of enlarged LLNs for increased LR rates, it is important to ensure sufficient awareness among all involved disciplines. Radiologists are the first to report the presence of LLNs to the multidisciplinary team (MDT) and it is vital that important characteristics, such as size and anatomical location, are examined during the diagnostic phase. The latest European Society of Gastrointestinal and Abdominal Radiology (ESGAR) consensus guidelines (2016) and the guidelines from the Society for Abdominal Radiologists (2018) both state that LLNs should be mentioned in MRI reports [2–4]. Furthermore, reporting of LLNs during MDT meetings is also essential to ensure appropriate decision making [5], considering that both radiation-oncologists and surgeons rely on radiology reports to develop their treatment plans after the MDT meeting.

This study aimed to evaluate how often radiologists explicitly stated the presence or absence of LLNs in primary and restaging MRI reports for patients with rectal carcinoma.

## Materials and methods

This study was approved by the medical ethics review board of Amsterdam UMC, location VUmc. Eligible patients were sent a letter describing the study and given the opportunity to opt out. Patients who had died before the study commenced were automatically included in the cohort.

This retrospective cohort study reviewed 322 patients who were treated for primary rectal carcinoma between January 2012 and December 2020 in Amsterdam UMC, location VUmc. All tumours with the possibility of spreading to the lateral compartments (≥ T2) and limited to within 12cm of the anus (as defined on MRI) were included. This broader selection than only ‘LARC’ helped ensure that all possible LLNs could be included. During the original review, all series and planes (axial, coronal and sagittal) were used. Patients with synchronous distant metastases, recurrent disease or without available MRI images/reports were excluded.

MRI reports were screened (TCS and YFLR) to determine if the radiologist mentioned the presence or absence of LLNs. Approximately 30–40 different radiologists had written these original reports. The reports were evaluated according to a predetermined list of terms associated with LLNs, such as extra-mesorectal, para-iliac, or lateral (Appendix 1). Ambiguous terms were evaluated by the researchers; if it was unclear whether mesorectal or LLNs were implied (e.g., locoregional), these were not considered as explicitly reporting on LLNs.

If an LLN was mentioned, the following characteristics were also extracted from the reports: short-axis size (in mm), anatomical compartment, malignant features (internal heterogeneity, irregular border, shape) and whether the LLN was considered suspicious. Whether and how these characteristics were reported was translated into an ‘overall score’. This overall score is a description of the total number of characteristics which were mentioned. For all cases, the primary MDT reports stored in electronic patient files were also reviewed to examine whether LLNs were discussed there.

**Fig. 1:**
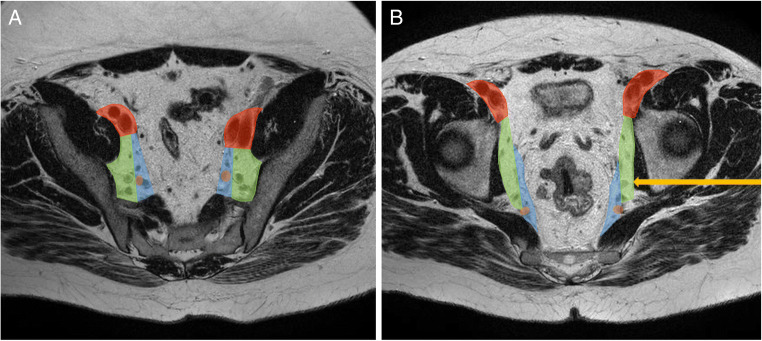
**A, B** Axial T2-MRI with colour atlas overlay depicting the lateral compartments. **A** Red—external iliac compartment, green—obturator compartment, blue—internal iliac compartment, orange spot—internal iliac artery, of which the lateral side of the main trunk forms the border between the obturator and internal iliac compartments. **B** Lateral lymph node, indicated with a yellow arrow

**Fig. 2:**
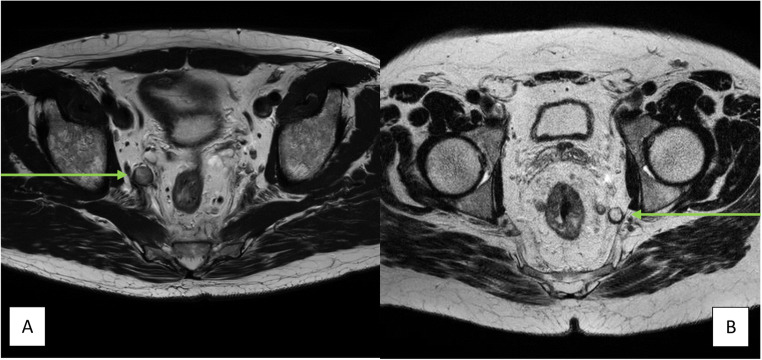
**A**, **B** Two examples of an enlarged and suspicious lateral lymph node on axial T2-MRI. Left (**A**): enlarged lateral lymph node (green arrow) located medial of the main trunk of the internal iliac artery and is therefore located in the internal iliac compartment. Right (**B**): enlarged lateral lymph node (green arrow) located caudal of where the main trunk of the internal iliac artery exits the pelvis and is therefore located in the obturator compartment

The MRI scans for all 202 patients were presented for expert re-review by an expert abdominal radiologist specialised in rectal cancer and lateral lymph nodes (8 years of experience), who independently scored the images. The short-axis size and anatomical location of LLNs were recorded according to definitions adhered to by Ogura et al [13] (Figs. 1A, B and 2A, B)

Statistical analyses were conducted using SPSS Statistics, version 26.0 (SPSS). The primary outcome was how many reports mentioned the presence or absence of LLNs. Secondary outcomes were which characteristics were mentioned, how many restaging reports mentioned LLNs and the incidence of LLNs identified during expert re-review compared to the original reports. All categorical data is presented as *n* and percentages. Continuous variables are presented as means with standard deviation. Chi-Squared test and Fisher’s exact tests were performed.

## Results

**Fig. 3:**
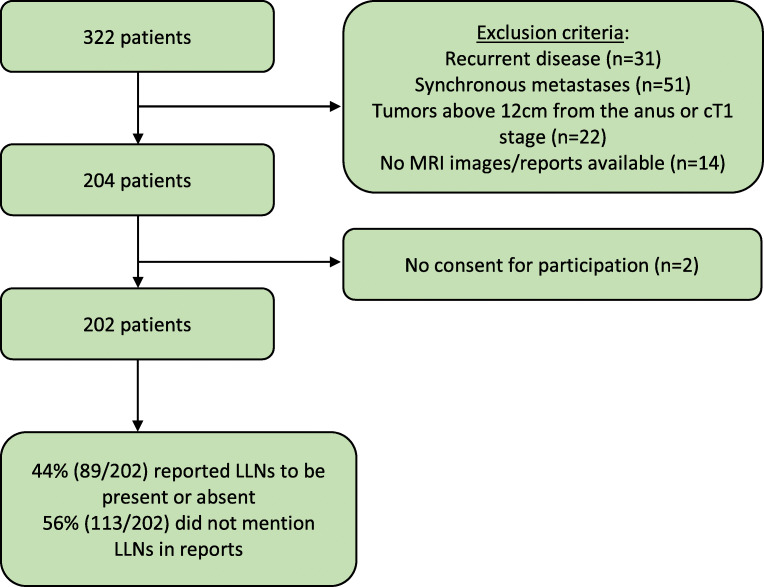
Study flowchart

A total of 202 patients were included for evaluation. Two patients opted out of the study (Fig. 3). Baseline characteristics are displayed in Table 1. Of the included patients, 126 (62.4%) were male with a mean age of 65.6 (SD 11.8) years.

**Table 1:** Baseline characteristics

	*N* (%)
Gender: male (%)	126 (62)
Age in years (mean, SD)	66 (12)
BMI (mean, SD)	25 (4.5)
ASA performance score: ASA 1–2 (%)	147 (73)
Previous pelvic surgery (%)	21 (10)
Previous malignancy (%)	31 (15)
**Mean height of tumour from landmark in cm (SD)**	
Anal verge	11.0 (0)
Anorectal junction	4.2 (3.3)
Ab ano/ani	5.2 (3.4)
Dentate line	1.8 (2.2)
Sphincter complex	3.1 (4.0)
Overall mean height	4.6 (3.5)
**Clinical T-stage (%)**	
Total cT2	41 (20)
Total cT3	121 (60)
cT3a	19 (9)
cT3b	38 19)
cT3c	10 (5)
cT3d	6 (3)
cT3 without specification	48 (24)
Total cT4	40 (20)
cT4a	8 (4)
cT4b	7 (4)
cT4 without specification	25 (12)
**Clinical N-stage (%)**	
N0	87 (43)
N1	76 (38)
N2	39 (19)
**Positive mesorectal fascia (%)**	
Yes	67 (33)
No	135 (67)
**Neoadjuvant treatment (%)**	
None	52 (26)
Short-course radiotherapy	74 (37)
Chemoradiotherapy	76 (38)
**Operation (%)**	
Local excision	25 (12)
Total mesorectal excision	104 (52)
Abdominal perineal resection	54 (27)
Proctocolectomy	1 (0.5)
Other	17 (8)

### Primary MRI-reports

The presence or absence of LLNs was mentioned in 89/202 (44%) primary MRI reports. In 43/89 (48%) cases, LLNs were reported as present, while the remaining 46/89 (52%) were reported as absent. For the 43 cases with present LLNs, 36 (84%) also mentioned a short-axis size, 15 (35%) an anatomical location and 11 (26%) stated the presence or absence of malignant features. When only considering cT3/4 tumours, or those located < 8 cm from the anorectal junction, rates of reporting LLNs were similarly low, 43% and 45% respectively.

Varying terms were used to describe LLNs: 33% reported ‘extra-mesorectal lymph nodes’, 32% ‘para-iliac’, 25% ‘outside the mesorectal fascia’, 5% ‘lateral lymph nodes’ and 5% as ‘lymph nodes in the obturator area’.

On the basis of the information gathered from the reports to determine an ‘overall score’, only one (1%) included information about the size (short-axis), anatomical location, the presence or absence of malignant features and whether or not the LLN was suspicious. The other 42 reports described varying degrees of these characteristics (Table 2).

**Table 2:** Primary MRI reports and overall scores

	*N* (%)	
Presence or absence of LLN mentioned	89/202 (44)	
Present	43/89 (48)	
Absent	46/89 (52)	
**Features described for present LLNs**		
Short-axis (SA) size mentioned	36/43 (84)	
Compartment mentioned	15/43 (35)	
Malignant features mentioned	11/43 (26)	
**Overall ‘score’ according to the degree of characteristics mentioned**		
*Characteristic(s) mentioned*	*N (%)*	*Examples of text*
LLN mentioned, visible, no further characteristics	5/43 (12)	*An extra-mesorectal lymph node*
LLN mentioned, including SA node size	16/43 (37)	*7 - mm extra - mesorectal lymph node*
LLN mentioned, including location	0 (0)	*Lymph node in the left internal iliac area*
LLN mentioned, including SA node size and location	5/43 (12)	*7 - mm lymph node in the left internal iliac area*
LLN mentioned, including SA node size and malignant features	5/43 (12)	*7 - mm heterogeneous lymph node*
LLN mentioned, including SA node size and consequences	1/43 (2)	*Suspicious 10-mm extra-mesorectal lymph node*
LLN mentioned, including location and malignant features	0 (0)	*Heterogeneous lymph node in left internal iliac area*
LLN mentioned, including location and consequences	1/43 (2)	*Suspicious lymph node in internal iliac area*
LLN mentioned, including malignant features and consequence	1/43 (2)	*Suspicious heterogenous extra-mesorectal lymph node*
LLN mentioned, including SA node size, location and malignant features	4/43 (9)	*7 - mm heterogenous lymph node in the internal iliac area*
LLN mentioned, including SA node size, location and consequences	4/43 (9)	*Suspicious 7 - mm lymph node in the internal iliac area*
LLN mentioned, including SA node size, location, malignant features and consequences	1/43 (2)	*Suspicious heterogeneous lymph node of 7 mm in the internal iliac area*

### Restaging MRI-reports

Half of the included patients (101/202, 50%) also underwent a restaging-MRI. In these reports, 34/101 (34%) mentioned the presence or absence of LLNs, of which 17/34 (50%) were reported as present. The SA size was mentioned in 13/17 (77%), anatomical location in 5/17 (29%) and 3/17 (18%) stated the presence or absence of malignant features.

For nine patients, an LLN was mentioned in the restaging report, but not in the primary report. In 17/43 (39.5%) cases where an LLN was described as present on the primary MRI, no mention was made in the restaging report (Table 3).

**Table 3:** Restaging MRI reports and overall scores

	*N* (%)	
Presence or absence of LLN mentioned	34/101 (34)	
Presence	17/34 (50)	
Absence	17/34 (50)	
**Features described for present LLNs**		
Short-axis (SA) size mentioned	13/17 (77)	
Compartment mentioned	5/17 (29)	
Malignant features mentioned	3/17 (18)	
**Overall ‘score’ according to the degree of characteristics mentioned**		
*Characteristic mentioned*	*N (%)*	*Examples of text*
LLN mentioned, visible, no further characteristics	2/17 (12)	*An extra-mesorectal lymph node*
LLN mentioned, including SA node size	7/17 (41)	*7 - mm extra-mesorectal lymph node*
LLN mentioned, including location	0/17 (0)	*Lymph node in the left internal iliac area*
LLN mentioned, including SA node size and location	2/17 (12)	*7 - mm lymph node in the left internal iliac area*
LLN mentioned, including SA node size and malignant features	1/17 (6)	*7 - mm heterogeneous lymph node*
LLN mentioned, including SA node size and consequences	0/17 (0)	*Suspicious 10-mm extra-mesorectal lymph node*
LLN mentioned, including location and malignant features	0/17 (0)	*Heterogeneous lymph node in left internal iliac area*
LLN mentioned, including location and consequences	1/17 (6)	*Suspicious lymph node in internal iliac area*
LLN mentioned, including malignant features and consequence	1/17 (6)	*Suspicious heterogenous extra-mesorectal lymph node*
LLN mentioned, including SA node size, location and malignant features	1/17 (6)	*7 - mm heterogenous lymph node in the internal iliac area*
LLN mentioned, including SA node size, location and consequences	1/17 (6)	*Suspicious 7 - mm lymph node in the internal iliac area*
LLN mentioned, including SA node size, location, malignant features and consequences	1/17 (6)	*Suspicious heterogeneous lymph node of 7 mm in the internal iliac area*

### Expert re-examination

For 188/202 patients, the images were re-examined. Fourteen cases were considered too poor to re-assess (10/14 were old scans from 2012/2013 and 4/14 due to artefacts from prosthetics). Enlarged LLNs (≥ 7 mm) were present in 17/188 (9%) patients. For two of these patients (2/17, 12%), the presence of LLNs was not mentioned in the primary reports. In the first case, nothing was mentioned in the report and for the second case ‘no lateral lymph nodes present’ was stated.

For the 15 cases where the anatomical location of an LLN was described, an agreement between expert re-review and primary report was only found in 2/15 (13%) cases. In the remaining cases, contradictory compartments were found. Five restaging cases mentioned an anatomical compartment, but for all five, expert re-review stated a different compartment compared to the original report.

### Influences on reporting

No significant correlation was found between the tumour (T)-stage and mentioning LLNs on the primary MRI report (*p* = .0495). A significant association was found for the N-stage; for those with a cN0-stage, 33.3% mentioned LLNs, while this was 52.2% for patients with a cN2-stage (*p* = 0.008). The height of the tumour did not influence how often LLNs were reported; for tumours < 8 cm versus 8–12cm, LLNs were reported as present/absent in 45% and 40% of cases respectively (*p* = 0.595). Based on the publication dates of the ESGAR and SAR guidelines, the incidence of reporting LLNs was examined according to time. This was 38% in reports before 2017 but increased to 52% from 2018 onwards (*p* = 0.042).

### Reports vs. primary MDT-meeting

Primary MRI reports described 43 patients with LLNs; 11/43 (26%) were also mentioned in MDT reports and described as suspicious. Three of these 11 LLNs (27%) were also suspicious in the radiology report, while the remaining eight cases were not suspicious according to the radiology report.

Another 3/43 (7%) were reported as not suspicious in the MDT report. For the remaining 29 cases, the LLN from the MRI report was not stated in the MDT report. Five of these 29 patients (17%) had an LLN which was described as suspicious in the primary MRI report.

### Oncological outcomes

Patients had a median follow-up time of 45 months (interquartile range 32–53 months) and 37 patients died during this period (18.3%). Thirty patients (14.9%) developed metachronous distant metastases.

LR occurred in 14 patients (6.9%) of which 3 were a lateral LR. For the first of these three cases, the LLN (12 mm left internal iliac) was mentioned in both the MRI - and MDT-reports. Patient underwent CRT after which the LLN was 9 mm. TME surgery (R0 resection) and left ‘node-picking’ followed. Two years later, an LLR formed in the left lateral pelvis. For the second patient, the LLN (7 mm left internal iliac) was also mentioned in both the MRI and MDT reports. After CRT (restaging: 6 mm), the patient underwent TME surgery (R0 resection). Five years later, the patient developed a left LLR.

The LLN (8.5 mm left internal iliac) of the third patient was not mentioned in the primary, restaging or MDT reports. Patient underwent CRT after which the LLN was still 8.5 mm, and an abdominoperineal resection followed (R0 resection). One year after surgery, a left LLR developed.

## Discussion

To our knowledge, this is the first study to investigate how often LLNs are mentioned in radiology reports and the results demonstrate significant underreporting. In less than half of the primary MRI reports, the presence or absence of LLNs was reported. Even less mentioned whether the enlarged LLN was present or absent in the restaging report. Furthermore, high variability was found for reporting important characteristics, with only 19% mentioning whether an LLN was considered suspicious or not and only 1% mentioning size and location as well as morphological criteria. Additionally, significant differences were found in the classification of anatomical compartments during expert re-review. In only 13% of primary cases, and in none of the restaging cases, consensus was observed between expert re-review and the primary report. Together, these results suggest a significant void in knowledge concerning LLNs, which needs to be addressed.

The results indicate not only limited awareness but also discrepancies between the original reports and expert re-review. Both of these facts may be explained by the fact that the majority of study participants are from before 2018, during which literature discussing LLNs was scarce. Since then, one article has published a colour atlas describing the exact borders of the lateral compartments [20] and other studies discussing LLNs have appeared. This growth in knowledge may have helped increase awareness, which is supported by the fact that this study also found a significant increase in reporting after 2018 compared to earlier years. Another important detail is that, until publication of a colour atlas by Ogura et al [20], there was no definitive guideline for the classification of lateral compartments and their anatomical borders. While the expert re-review specifically followed the outline of anatomical borders displayed in the colour atlas, the original reports would have been based on personal experience, allowing for vast discrepancies when comparing the two.

It is furthermore important to discuss the oncological implications of this study. For one of the three LLRs in this cohort, LLNs were not mentioned in primary, restaging or MDT reports and an LLR occurred just one year later. Another underwent ‘node-picking’, in which only the suspicious area, and not all of the lymphatic tissue, is removed. Though sparingly investigated, two small retrospective series of 12 and 30 patients indicate that node-picking is insufficient in decreasing the LR risk [13, 25, 26]. Furthermore, all three cases display LLNs larger than the reference values from Ogura et al, suggesting an increased LR risk requiring additional treatment [13, 20]. As described in the ‘Introduction’ section, an LLND may be essential if LLNs remain enlarged after CRT. It is therefore important to reflect not only on the necessity for correct reporting and communication, but also ensuring optimal treatment as a result of this improved awareness.

In recognition of the limited awareness of LLNs in MRI reports and considering the clinical implications that LLNs hold [13, 16, 17, 19, 20, 27, 28], this underreporting is substantial and needs to be addressed. This is especially so when considering that referring physicians rely on radiology reports for their subsequent treatment decisions. Education and training will be essential, while the introduction of templates should be discussed. A recent study in the UK demonstrated significant improvements in reporting ‘difficult’ elements of radiology, such as tumour deposits or extra-mural vascular invasion, from around 50 to almost 100% after introducing radiology templates [29]. While they did not specifically investigate LLNs, it is possible that templates would also improve the reporting LLNs. Although no study has specifically investigated reporting of LLNs, Sahni et al found that the proportion of radiology reports classified as ‘optimal’ for rectal cancer staging improved from 38.5 to 70.4% after introducing templates [30] and another study found similar improvements in the quality of reports (scored by independent panel as 4/10 before and 7/10 after implementing templates) [31]. One study also found that referring physicians, such as surgeons, had a preference for structured reports as aid for their surgical planning (94% preferred structured reports) [32]. Considering these improvements, the merit of introducing templates should be considered. Furthermore, training should be considered for the entire multidisciplinary team, not only radiologists, given the fact that reportedly present LLNs were still often not discussed during MDT meetings [33]. It may further be useful to create clear (international) guidelines. This would provide an overview of all current evidence regarding the importance of LLNs, along with proposed terminology to improve communication and clarity, and detail anatomical borders and short-axis thresholds considered as suspicious to improve consensus.

Support for improving awareness and reporting might also be found in other methods. For example, Bedrikovetski et al found that deep-learning models and radiomics outperformed radiologists for staging colorectal cancers (0.91 deep-learning, 0.79 radiomics and 0.64 radiologist) [34]. Alternatively, Minn et al created an algorithm to highlight errors made in 82,353 radiology reports over a 4-month period [35]. This tool improved complete reporting and allowed for a quick correction of mistakes, resulting in an overall reduction of errors. By introducing templates where it is necessary to complete a section on the presence/absence of LLNs, together with software that detects incomplete reports, it would become difficult to omit this necessary information from reports. In this manner, AI may in the future become essential in supporting and assisting radiologists in their diagnostic process [36].

There are several limitations related to this study. Importantly, this study is only able to investigate how many reports mention the presence or absence of LLNs, which is not a perfect translation to awareness. It is possible that radiologists did look for LLNs, so were ‘aware’, but did not write down their findings, positive or negative. Additionally, the retrospective design of this study is limiting. There was significant heterogeneity present between MRI reports, how the reports were structured and formulated, and patients had to be excluded due to missing images and/or reports. This was particularly so for earlier years, potentially forming a selection bias. This was also a single-centre study, limiting the number of cases to investigate and the external validity. A similar, multicentre study including international centres with larger cohorts would be interesting.

## Conclusion

This study revealed that the presence or absence of LLNs was mentioned in less than half of the primary MRI reports in patients treated for rectal cancer between 2012 and 2020 in a tertiary referral centre. Though improving with time, significant underreporting is present, demonstrating a substantial lack of awareness and knowledge regarding LLNs. Considering the clinical implications of enlarged LLNs, MRI reports should include all necessary information. This could be improved by the introduction of templates and increased knowledge regarding the significance of LLNs for oncological outcomes.

## Appendix 1: Pre-determined list of terms accepted as referring to LLNs

- Extra-mesorectal
- Lateral lymph nodes
- (Lymph nodes) in obturator area
- (Lymph nodes) in iliac area
- Para-iliac
- Para-obturator
- (Lymph node) along internal iliac artery
- (Lymph node) along external iliac artery
- (Lymph node) along obturator artery
- Outside the mesorectal fat
- Outside the mesorectal fascia
- Outside the mesorectum

Any other terms, such as ‘lymph node’, ‘node’ or ‘regional lymph node’ were considered ambiguous and not clearly referring to LLNs.

## Abbreviations

CRT: Chemoradiotherapy
LARC: Locally advanced rectal cancer
LLN(s): Lateral lymph node(s)
LLND: Lateral lymph node dissection
LLR: Lateral local recurrence
LR: Local recurrence
MDT: Multidisciplinary team
MRI: Magnetic resonance imaging
TME: Total mesorectal excision

## Declarations

### Guarantor

The scientific guarantor of this publication is Miranda Kusters.

### Statistics and biometry

No complex statistical methods were necessary for this paper.

### Methodology

• Retrospective cohort

• observational

• performed at one institution

## References

[1] Beets-Tan RG, Beets GL (2004). Rectal cancer: review with emphasis on MR imaging. Radiology..

[2] Beets-Tan RG, Lambregts DM, Maas M (2013). Magnetic resonance imaging for the clinical management of rectal cancer patients: recommendations from the 2012 European Society of Gastrointestinal and Abdominal Radiology (ESGAR) consensus meeting. Eur Radiol.

[3] Beets-Tan RGH, Lambregts DMJ, Maas M (2018). Magnetic resonance imaging for clinical management of rectal cancer: updated recommendations from the 2016 European Society of Gastrointestinal and Abdominal Radiology (ESGAR) consensus meeting. Eur Radiol.

[4] Gollub MJ, Arya S, Beets-Tan RG (2018). Use of magnetic resonance imaging in rectal cancer patients: Society of Abdominal Radiology (SAR) rectal cancer disease-focused panel (DFP) recommendations 2017. Abdom Radiol (NY).

[5] Horvat N, Carlos Tavares Rocha C, Clemente Oliveira B, Petkovska I, Gollub MJ (2019). MRI of rectal cancer: tumor staging, imaging techniques, and management. Radiographics..

[6] Pramateftakis MG, Kanellos D, Tekkis PP, Touroutoglou N, Kanellos I (2012) Rectal cancer: multimodal treatment approach. Int J Surg Oncol. 10.1155/2012/279341 10.1155/2012/279341 PMC3447353 23008766

[7] Swedish Rectal Cancer Trial, Cedermark B, Dahlberg M et al (1997) Improved survival with preoperative radiotherapy in resectable rectal cancer. N Engl J Med 336(14):980–987 10.1056/NEJM199704033361402 9091798

[8] Kapiteijn E, Marijnen CA, Nagtegaal ID (2001). Preoperative radiotherapy combined with total mesorectal excision for resectable rectal cancer. N Engl J Med.

[9] Kodeda K, Johansson R, Zar N et al (2015) Time trends, improvements and national auditing of rectal cancer management over an 18-year period. Colorectal Dis 17(9). 10.1111/codi.13060 10.1111/codi.13060 26155848

[10] Steup WH, Moriya Y, van de Velde CJ (2002). Patterns of lymphatic spread in rectal cancer. A topographical analysis on lymph node metastases. Eur J Cancer.

[11] Kim JH, Beets GL, Kim MJ, Kessels AG, Beets-Tan RG (2004). High-resolution MR imaging for nodal staging in rectal cancer: are there any criteria in addition to the size?. Eur J Radiol.

[12] Brown G, Richards CJ, Bourne MW (2003). Morphologic predictors of lymph node status in rectal cancer with use of high-spatial-resolution MR imaging with histopathologic comparison. Radiology..

[13] Ogura A, Konishi T, Cunningham C (2019). Neoadjuvant (Chemo)radiotherapy with total mesorectal excision only is not sufficient to prevent lateral local recurrence in enlarged nodes: results of the multicenter lateral node study of patients with Low cT3/4 rectal cancer. J Clin Oncol.

[14] Georgiou P, Tan E, Gouvas N (2009). Extended lymphadenectomy versus conventional surgery for rectal cancer: a meta-analysis. Lancet Oncol.

[15] Peacock O, Chang GJ (2020). The landmark series: management of lateral lymph nodes in locally advanced rectal cancer. Ann Surg Oncol.

[16] Kim MJ, Kim TH, Kim DY (2015). Can chemoradiation allow for omission of lateral pelvic node dissection for locally advanced rectal cancer?. J Surg Oncol.

[17] Kim TG, Park W, Choi DH (2014). Factors associated with lateral pelvic recurrence after curative resection following neoadjuvant chemoradiotherapy in rectal cancer patients. Int J Colorectal Dis.

[18] Akiyoshi T, Ueno M, Matsueda K (2014). Selective lateral pelvic lymph node dissection in patients with advanced low rectal cancer treated with preoperative chemoradiotherapy based on pretreatment imaging. Ann Surg Oncol.

[19] Williamson JS, Quyn AJ, Sagar PM (2020). Rectal cancer lateral pelvic sidewall lymph nodes: a review of controversies and management. Br J Surg.

[20] Ogura A, Konishi T, Beets GL et al (2019) Lateral nodal features on restaging magnetic resonance imaging associated with lateral local recurrence in low rectal cancer after neoadjuvant chemoradiotherapy or radiotherapy. JAMA Surg 154(9). 10.1001/jamasurg.2019.2172 10.1001/jamasurg.2019.2172 PMC6613303 31268504

[21] Christou N, Meyer J, Toso C, Ris F, Buchs NC (2019). Lateral lymph node dissection for low rectal cancer: is it necessary?. World J Gastroenterol.

[22] Ito M, Kobayashi A, Fujita S et al (2018) Urinary dysfunction after rectal cancer surgery: results from a randomized trial comparing mesorectal excision with and without lateral lymph node dissection for clinical stage II or III lower rectal cancer (Japan Clinical Oncology Group Study, JCOG0212). Eur J Surg Oncol 44(4):463–468 10.1016/j.ejso.2018.01.015 29428473

[23] Saito S, Fujita S, Mizusawa J et al (2016) Male sexual dysfunction after rectal cancer surgery: results of a randomized trial comparing mesorectal excision with and without lateral lymph node dissection for patients with lower rectal cancer: Japan Clinical Oncology Group Study JCOG0212. Eur J Surg Oncol 42(12):1851–1858 10.1016/j.ejso.2016.07.010 27519616

[24] Fujita S, Mizusawa J, Kanemitsu Y et al (2017) Mesorectal excision with or without lateral lymph node dissection for clinical stage II/III lower rectal cancer (JCOG0212): a multicenter, randomized controlled, noninferiority trial. Ann Surg 266(2):201–207 10.1097/SLA.0000000000002212 28288057

[25] Kim YI, Jang JK, Park IJ (2020). Lateral lymph node and its association with distant recurrence in rectal cancer: a clue of systemic disease. Surg Oncol.

[26] Sluckin TC, Hazen SJA, Kusters M (2021). From “East vs West” towards international multidisciplinary collaboration: an appraisal of lateral lymph nodes in rectal cancer. Ann Gastroenterol Surg.

[27] Kim MJ, Hur BY, Lee ES et al (2018) Prediction of lateral pelvic lymph node metastasis in patients with locally advanced rectal cancer with preoperative chemoradiotherapy: Focus on MR imaging findings. PLoS One 13(4). 10.1371/journal.pone.0195815 10.1371/journal.pone.0195815 PMC5897011 29649321

[28] Kim TH, Jeong SY, Choi DH (2008). Lateral lymph node metastasis is a major cause of locoregional recurrence in rectal cancer treated with preoperative chemoradiotherapy and curative resection. Ann Surg Oncol.

[29] Brown PJ, Rossington H, Taylor J (2019). Standardised reports with a template format are superior to free text reports: the case for rectal cancer reporting in clinical practice. Eur Radiol.

[30] Sahni VA, Silveira PC, Sainani NI, Khorasani R (2015). Impact of a structured report template on the quality of MRI reports for rectal cancer staging. AJR Am J Roentgenol.

[31] Tersteeg JJC, Gobardhan PD, Crolla R (2018). Improving the quality of MRI reports of preoperative patients with rectal cancer: effect of national guidelines and structured reporting. AJR Am J Roentgenol.

[32] Nörenberg D, Sommer WH, Thasler W (2017). Structured reporting of rectal magnetic resonance imaging in suspected primary rectal cancer: potential benefits for surgical planning and interdisciplinary communication. Invest Radiol.

[33] Waite S, Scott JM, Drexler I (2018). Communication errors in radiology - pitfalls and how to avoid them. Clin Imaging.

[34] Bedrikovetski S, Dudi-Venkata NN, Maicas G et al (2021) Artificial intelligence for the diagnosis of lymph node metastases in patients with abdominopelvic malignancy: a systematic review and meta-analysis. Artif Intell Med 113. 10.1016/j.artmed.2021.102022 10.1016/j.artmed.2021.102022 33685585

[35] Minn MJ, Zandieh AR, Filice RW (2015). Improving Radiology Report Quality by Rapidly Notifying Radiologist of Report Errors. J Digit Imaging.

[36] Thrall JH, Li X, Li Q (2018). Artificial intelligence and machine learning in radiology: opportunities, challenges, pitfalls, and criteria for success. J Am Coll Radiology.

