# Correction to: The awareness of radiologists for the presence of lateral lymph nodes in patients with locally advanced rectal cancer: a single-centre, retrospective cohort study

**DOI:** 10.1007/s00330-022-08902-4

**Published:** 2022-06-30

**Authors:** T. C. Sluckin, Y. F. L. Rooker, S. Q. Kol, S. J. A. Hazen, J. B. Tuynman, J. Stoker, P. J. Tanis, K. Horsthuis, M. Kusters

**Affiliations:** 1 Department of Surgery, Amsterdam UMC location Vrije Universiteit Amsterdam, De Boelelaan 1117, Amsterdam, the Netherlands; 2 Cancer Center Amsterdam, Treatment and Quality of Life, Amsterdam, the Netherlands; 3 Cancer Center Amsterdam, Imaging and Biomarkers, Amsterdam, the Netherlands; 4 Department of Radiology and Nuclear Medicine, Amsterdam UMC location Vrije Universiteit Amsterdam, de Boelelaan 1117, Amsterdam, the Netherlands; 5 Department of Radiology and Nuclear Medicine, Amsterdam UMC location University of Amsterdam, Meibergdreef 9, Amsterdam, the Netherlands; 6 Department of Surgical Oncology and Gastrointestinal Surgery, Erasmus MC, Rotterdam, the Netherlands; 7 Department of Surgery, Amsterdam UMC location University of Amsterdam, Meibergdreef 9, Amsterdam, the Netherlands

**Correction to: European Radiology**

10.1007/s00330-022-08840-1

The original version of this article, published on 18 May 2022, unfortunately contained some mistakes. The affiliations were incorrectly rearranged during the typesetting stage. They are now corrected in this paper.

The original article has been corrected.

